# Maternal–foetal immune rejection: parallels between placental CHI and allograft rejection^†^

**DOI:** 10.1002/path.6459

**Published:** 2025-07-29

**Authors:** Panicos Shangaris, María Teresa Martín Monreal

**Affiliations:** ^1^ Department of Women and Children's Health, School of Life Course Sciences, Faculty of Life Sciences & Medicine King's College London London UK; ^2^ Harris Birthright Research Centre for Foetal Medicine, King's College Hospital London UK; ^3^ Peter Gorer Department of Immunobiology, School of Immunology & Microbial Sciences, Faculty of Life Sciences & Medicine King's College London London UK

**Keywords:** chronic histiocytic intervillositis, maternal–foetal rejection, placenta, antibody‐mediated rejection, immunotherapy, transplant immunology, pregnancy loss, immune tolerance

## Abstract

Chronic histiocytic intervillositis (CHI) is a rare placental inflammatory lesion increasingly viewed through maternal–foetal immune rejection. In this invited commentary, we discuss how a recent study bolsters the paradigm that CHI represents maternal immune rejection of the semi‐allogeneic foetus, analogous to antibody‐mediated rejection in organ transplantation. We highlight the shared histological and molecular features between CHI and kidney allograft rejection, including macrophage‐dominated inflammation, complement activation, and interferon‐gamma‐driven gene expression signatures and explore the implications of this common pathogenesis for clinical practice. Recognising CHI as an alloimmune process opens new avenues for early prediction, diagnosis, and intervention. We particularly emphasise the need for early identification of CHI, even in a first pregnancy, where no prior obstetric history exists to raise suspicion. Finally, we outline how transplant immunotherapy principles (e.g. immunosuppression and immune modulation) could transform the management of CHI, and we call for forward‐looking research that bridges immune pathology, maternal–foetal medicine, and translational therapeutics to improve pregnancy outcomes. © 2025 The Author(s). *The Journal of Pathology* published by John Wiley & Sons Ltd on behalf of The Pathological Society of Great Britain and Ireland.

## Chronic histiocytic intervillositis and the maternal–foetal rejection hypothesis

This commentary relates to a recent article published in this journal on the similarity of features between chronic histiocytic intervillositis (CHI) and antibody‐mediated rejection of kidney allografts [1]. CHI is a rare placental lesion characterised by an abnormal, predominantly macrophagic infiltrate in the intervillous space of the placenta [2]. First described in 1987, CHI is diagnosed when ≥5% of the intervillous space is occupied by inflammatory cells (≥80% of which are CD68^+^ macrophages) in the absence of infection [3]. CHI is associated with adverse outcomes, including severe foetal growth restriction, miscarriage, and stillbirth, and notably has a high recurrence rate in subsequent pregnancies [4, 5]. Unfortunately, because CHI is rare (~6 in 10,000 pregnancies) and often identified only after pregnancy loss or placental examination, there are currently limited guidelines for diagnosing this condition in an index pregnancy [4]. This lack of early diagnostic strategies contributes to its poor perinatal outcomes and the challenge of prevention.

Pregnancy is usually a state of immune tolerance: the foetus and placenta are semi‐allogeneic (sharing paternal antigens), yet typically evade attack by the maternal immune system [6]. The placenta achieves this immune privilege through multiple mechanisms (limited expression of polymorphic human leukocyte antigen [HLA] class I/II molecules, local immune regulatory cells, and immunosuppressive factors). CHI represents a breakdown of this immunological tolerance—the maternal immune system ‘rejects’ the placenta as if it were a foreign graft [7]. Indeed, CHI may represent a host‐versus‐graft reaction akin to transplant rejection [8]. This maternal–foetal immune rejection hypothesis has gained traction, as numerous parallels have emerged between CHI and antibody‐mediated rejection of transplanted organs [1]. For example, several cases of CHI have been linked to maternal donor‐specific antibodies against paternal HLA antigens, with complement split‐product C4d deposition in the placenta as a telltale marker [1, 7]. Such features mirror the well‐known *Banff criteria* for antibody‐mediated rejection (AMR) in kidney allografts, where recipient antidonor HLA antibodies, C4d positivity, and macrophage‐rich inflammation portend graft failure [1]. These clinicopathological analogies led Benachi *et al* to propose that CHI is essentially an alloimmune placental rejection phenomenon [7]. The study by Albersammer *et al* in *The Journal of Pathology* provides compelling new evidence in support of this paradigm [1].

## Shared pathology between placental CHI and allograft rejection

Albersammer *et al* undertook an in‐depth histologic and molecular analysis of CHI, explicitly drawing comparisons to antibody‐mediated kidney transplant rejection [1]. In their cohort of 12 CHI‐affected placentas (all positive for C4d and foetal‐specific HLA antibodies) *versus* five control placentas, they applied multiplex immunofluorescence and a transplant‐relevant gene expression panel (NanoString B‐ HOT) to characterise the immune infiltrate and molecular milieu. Placental lesions in CHI exhibited a predominance of M1‐polarised macrophages (CD68^+^/CD206^−^) occupying the intervillous space, along with a notable increase in CD8^+^ T lymphocytes. CD4^+^ T cells and natural killer cells were scarce; moreover, M1 macrophages and CD8^+^ T cells were absent in all control samples. This immune profile is reminiscent of acute antibody‐mediated rejection rather than chronic rejection, given the prominence of macrophages, CD8^+^ T cells, and complement activation. Furthermore, the CHI placentas demonstrated upregulation of HLA class II in gene profiling and at the protein level (immunohistochemistry), alongside a robust interferon‐γ (IFN‐γ)–driven cytokine signature—molecular hallmarks that parallel the transcriptomic profile of rejecting kidney allografts. Notably, IFN‐γ is a key cytokine in allograft rejection, promoting HLA upregulation and macrophage activation; its prominence in CHI underscores the analogous Th1‐skewed inflammatory environment at the maternal–foetal interface. These shared histological and molecular features reinforce that CHI and transplant rejection have a common immunopathogenesis [1]. In both scenarios, alloantigen recognition (paternal in CHI, donor in transplants) leads to antibody‐ and complement‐mediated attack, with downstream recruitment of mononuclear phagocytes and T cells that orchestrate tissue damage. Remarkably, placental tissue in CHI can fulfil the diagnostic criteria originally defined for solid‐organ graft rejection [1]. This validates the maternal–foetal immune rejection hypothesis and opens the door to applying decades of transplantation immunology knowledge to better understand and manage CHI. Table 1 illustrates the key parallels between maternal–foetal placental and allograft rejection, from immune mechanisms to potential therapeutic targets.

**Table 1:** Parallels between maternal–foetal placental rejection (CHI) and transplant allograft rejection

Feature	Maternal–foetal placental rejection (CHI)	Transplant allograft rejection
(a) Antibody Response	Maternal anti‐foetal (paternal) HLA antibodies	Donor‐specific HLA antibodies
Complement Activation	Yes – triggers C4d deposition in placenta	Yes – triggers C4d deposition in transplanted organ
(b) Immune Cell Infiltrates	Activated macrophages and CD8+ T cells in intervillous space	Activated macrophages and CD8+ T cells in graft tissue
(c) Inflammatory Milieu	IFN‐γ–rich environment, HLA Class II upregulation at maternal–foetal interface	IFN‐γ–driven cytokine storm, MHC upregulation in graft
(d) Diagnostic Biomarkers	C4d immunostaining, IFN‐γ signatures	C4d immunostaining, IFN‐γ signatures
Therapeutic Targets	Steroids, IVIG, TNF inhibitors, complement inhibitors	Steroids, IVIG, TNF inhibitors, complement inhibitors
Pathological Outcome	Placental tissue damage, growth restriction, pregnancy loss	Graft tissue damage, graft failure

## Implications for early prediction and diagnosis

A major challenge is that CHI often comes to light only after significant damage is done, typically after pregnancy loss or by pathological examination of a placenta from a complicated pregnancy. By then, the opportunity to intervene has passed. Given CHI's high recurrence risk and devastating outcomes [7], there is a pressing need to develop methods for early identification of at‐risk pregnancies. While CHI often manifests in subsequent pregnancies due to sensitisation during an earlier, clinically uneventful pregnancy, it is crucial to explore strategies that might identify developing alloimmune risk even in primigravidas – recognising that truly early‐onset CHI may be rare. Particularly for primigravidas, although rare, monitoring for *de novo* immune activation may help identify pregnancies at risk, especially when other markers of placental dysfunction emerge. Recognising CHI as an alloimmune disorder suggests several forward‐looking strategies for prediction and diagnosis.

### Immunological screening

As transplant candidates undergo panel reactive antibody testing and donor‐specific crossmatching, one could envision screening pregnant mothers for anti‐paternal HLA antibodies early in gestation. If a woman is found to have strong reactivity against the foetus's HLA antigens (so‐called *foetal‐specific antibodies*), this could raise concern for a CHI‐like process developing. However, it is important to note that not all women with foetal‐specific antibodies go on to develop CHI. As shown previously [4], the presence of these antibodies is not exclusive to CHI and can be found in uncomplicated pregnancies, highlighting the need for additional markers to improve specificity [4]. Recent studies [1] using techniques like ‘virtual crossmatch’ in CHI have begun to explore this, although the findings are mixed regarding the prevalence of maternal anti‐HLA in CHI *versus* normal pregnancies [4]. Timing may be critical; antibodies might emerge or spike only after placental injury is well underway [4]. Therefore, serial monitoring of maternal alloantibodies during pregnancy could be informative, especially in women with a history of CHI.

### Molecular and imaging biomarkers

The gene expression signature characterised by heightened HLA class II and inflammatory cytokines [1] raises the question of whether noninvasive biomarkers could signal a developing CHI. For instance, elevated levels of IFN‐γ or related cytokines in maternal serum or cell‐free RNA transcripts indicative of placental stress might be early warning signs. Advanced imaging techniques (e.g. Doppler ultrasound of uteroplacental circulation or novel MRI methods) are not specific to CHI. Still, they could detect downstream effects such as impaired perfusion or inflammation‐related placental changes earlier than gross anatomy. Research is needed to identify a combination of immunologic and imaging markers that could prompt early diagnostic evaluation.

### Placental pathology vigilance

When there is any hint of abnormal placental function, such as unexplained severe growth restriction or unusual findings on prenatal surveillance, clinicians should be vigilant about considering a placental pathology like CHI. In practice, a definitive CHI diagnosis requires placental histology. Thus, in cases of unexplained late first‐trimester or second‐trimester loss or severe foetal growth restriction, sending the placenta for expert pathological examination is crucial. Increased awareness of CHI among pathologists and obstetricians will improve detection. Early postpartum diagnosis in an index pregnancy, while too late to save that pregnancy, is valuable, as it allows planning of preventive strategies in subsequent pregnancies [7]. Additionally, systematic use of C4d immunostaining in placental specimens – especially in cases of unexplained foetal growth restriction or loss – may aid in identifying immune‐mediated pathology and prompt consideration of CHI.

In summary, treating CHI as a form of immune rejection incentivises the development of predictive tests akin to transplant medicine, from antibody screening to molecular assays, to identify cases as early as possible. Particularly for primigravidas with no prior red flags, such tools could be lifesaving, allowing interventions to commence before irreversible placental damage occurs.

## Implications for treatment and immunotherapy

Perhaps the most promising aspect of the maternal–foetal immune rejection paradigm is the potential to translate immunotherapeutic approaches from transplant medicine to CHI. Suppose the maternal immune system is indeed rejecting the placenta in CHI. In that case, it stands to reason that modulating that immune attack could improve pregnancy outcomes, just as immunosuppression can salvage an allograft. Treatment experience in CHI has been limited to case reports and small series, but a recent systematic review offers some guidance [2]. In an analysis of over 659 CHI pregnancies, various immunomodulatory treatments were reported – most commonly low‐dose aspirin, corticosteroids, low‐molecular‐weight heparin, hydroxychloroquine, and tumour necrosis factor (TNF) inhibitors like adalimumab – to dampen maternal inflammation and improve placental outcomes [2]. The findings suggested a trend toward improved live birth rates with treatment, but the improvement was not statistically significant, given the rarity and heterogeneity of cases. Notably, that review reinforced that more severe CHI lesions correlate with higher odds of pregnancy loss, underscoring that effective therapy must ideally prevent the lesion from becoming extensive [2].

Encouragingly, some individual reports have shown promise with aggressive immunotherapy. For instance, intravenous immunoglobulin (IVIG) – used to quell antibody‐mediated processes – has yielded positive outcomes in recurrent CHI, as highlighted in the first reported successful use of IVIG for CHI [6]. IVIG may work by neutralising pathogenic antibodies and broadly modulating the immune response, and it has an acceptable safety profile. Other therapies borrowed from transplant and autoimmunity realms merit exploration in CHI: plasmapheresis or immunoadsorption to remove maternal allo‐antibodies; high‐dose corticosteroids to suppress macrophage, and T‐cell activation; and targeted biologics such as complement inhibitors (e.g. eculizumab, which is pregnancy‐compatible) to prevent C4d‐mediated damage, or B‐cell‐depleting agents (e.g. rituximab) to reduce antibody production. Indeed, a recent case series noted that immunomodulatory therapy could reduce the severity of placental lesions in CHI [9], hinting that timely intervention might translate to better foetal outcomes. Furthermore, IL‐1 blockade using Anakinra, a treatment approved in pregnancy for certain auto‐inflammatory conditions, has emerged as a promising option. Inflammasome‐targeted therapy may also prevent adverse perinatal outcomes in recurrent CHI, warranting further exploration in clinical trials [10].

Crucially, any immunotherapy for CHI must balance efficacy with safety for mother and foetus. Many immunosuppressive drugs used in transplant patients are contraindicated or risky in pregnancy. However, some agents (like TNF inhibitors, IL‐1 antagonists, or hydroxychloroquine) have been used in pregnant patients with autoimmune diseases and could be repurposed for CHI. The translational path forward should include prospective trials or registries for CHI despite the challenges of studying such a rare condition [2]. Collaborative efforts, possibly leveraging global networks, will be needed to gather enough cases to test therapies systematically. In the meantime, a reasonable approach in practice for women with a history of CHI is combination therapy aiming to counteract the alloimmune response: for example, low‐dose aspirin and heparin for their anti‐inflammatory and pro‐perfusion effects, plus prednisone and hydroxychloroquine to attenuate immune activation [2]. The addition of IVIG or other agents can be considered in refractory cases [6]. While the paradigm of maternal–foetal rejection explains a subset of CHI cases, particularly those with C4d and FSA positivity, it is important to acknowledge heterogeneity. Some CHI placentas do not show C4d deposition or foetal‐specific antibodies, indicating that other, potentially non‐alloimmune, mechanisms may contribute to the pathogenesis in these cases. Personalisation based on each patient's immunological profile is an appealing concept that echoes precision medicine in transplantation.

## Conclusion and future perspectives

The work by Albersammer *et al* [1] crystallises an exciting convergence between placental pathology and transplant immunology. By providing concrete histologic and molecular evidence that CHI and antibody‐mediated allograft rejection share a common pathogenesis [1, 7], this study validates the maternal–foetal immune rejection hypothesis and lays a foundation for cross‐disciplinary innovation. A placenta suffering from CHI can now be seen, in a sense, as a ‘rejected graft’ – a perspective that not only enriches our scientific understanding but also carries profound translational implications. We anticipate a more forward‐looking, proactive approach to CHI that emphasises early detection of maternal alloimmune risk and the prompt initiation of therapies to protect the placenta, much like we would protect a transplanted organ. This could involve closer monitoring of pregnancies deemed at‐risk, application of novel diagnostic tools, and the judicious use of immunomodulators to promote tolerance at the maternal–foetal interface.

Bridging the gap between immunopathology and maternal–foetal medicine will require concerted efforts. Obstetricians, immunologists, transplant specialists, and pathologists must collaborate to unravel the immunology of CHI further, investigate safe therapeutic regimens, and perhaps identify preventive strategies for women at high risk (previous CHI or known anti‐foetal HLA sensitisation). The ripple effects of this line of research could extend to other obstetric syndromes thought to involve maladaptive immunity (recurrent miscarriage, severe preeclampsia, etc.). CHI, with its dramatic pathology and clear immunologic footprint, is an ideal starting point. In summary, treating CHI as a model of maternal–foetal immune rejection not only deepens our understanding of this rare disease but also exemplifies how insights from one field (transplantation) can catalyse advances in another (reproductive medicine). By embracing this paradigm, we move closer to predictive, preventative, and personalised strategies to ensure that mothers and babies are spared the tragic consequences of an unrecognised immune conflict.

Both scenarios share standard mechanisms and targets: (a) Maternal anti‐foetal (paternal) HLA antibodies in CHI *versus* donor‐specific HLA antibodies in transplant rejection, activating the complement system and yielding C4d deposition in the affected tissue. (b) Immune cell infiltrates are dominated by activated macrophages and CD8^+^ T cells, causing tissue damage in the placenta (intervillous space), just as in rejecting kidneys. (c) An IFN‐γ–rich inflammatory milieu and upregulation of HLA Class II molecules at the maternal–foetal interface, mirroring the cytokine storm and MHC upregulation in organ rejection. (d) Overlapping biomarkers and therapeutic targets, such as C4d immunostaining as a diagnostic marker in both CHI and graft rejection, and the use of immunosuppressive therapies (steroids, IVIG, TNF inhibitors, complement inhibitors) aimed at the antibody–complement–macrophage axis in both conditions. This comparative framework highlights how insights from transplant immunotherapy could inform future treatments for CHI.

## Author contributions statement

PS conceived the concept of the commentary, reviewed the literature, and wrote the article. MTMM reviewed and edited the article. All authors approved the final version of the article.

## Data Availability

Data sharing is not applicable to this article as no new data were created or analysed in this study.
